# Long term effects of self-determination theory and motivational interviewing in a web-based physical activity intervention: randomized controlled trial

**DOI:** 10.1186/s12966-015-0262-9

**Published:** 2015-08-18

**Authors:** Stijn AH Friederichs, Anke Oenema, Catherine Bolman, Lilian Lechner

**Affiliations:** Open University of the Netherlands, Faculty of Psychology and Educational Sciences, P.O. box 2960, 6401 DL Heerlen, The Netherlands; Department of Health Promotion, Maastricht University, P.O. Box 616, 6200 MD Maastricht, The Netherlands

**Keywords:** Physical activity, Motivational interviewing, Self-determination theory

## Abstract

**Background:**

Our main objective in the current study was to evaluate the long-term effectiveness (12 months from baseline) of I Move (a web-based computer tailored physical activity intervention, based on self-determination theory and motivational interviewing). To this end, we compared I Move to a web-based computer tailored physical activity intervention based on traditional health behavior theories (Active Plus), and to a no-intervention control group. As a secondary objective, the present study aimed to identify participant characteristics that moderate the long term effects of I Move and Active Plus.

**Methods:**

A randomized controlled trial was conducted, comparing three research conditions: 1) the I Move condition, participants in this condition received I Move; 2) the Active Plus condition, participants in this condition received Active Plus; 3) the control condition; participants in this condition received no intervention and were placed on a waiting list. Main outcome measures were weekly minutes of moderate to vigorous physical activity and weekly days with minimal 30 min of physical activity. All measurements were taken by web-based questionnaires via the study website. Intervention effects were analyzed using multilevel linear regression analyses.

**Results:**

At 12 months from baseline, I Move was found to be effective in increasing weekly minutes of moderate to vigorous physical activity (ES = .13), while Active Plus was not. In contrast, Active Plus was found to be effective in increasing weekly days with ≥ 30 min PA at 12 months (ES = .11), while I Move was not. No moderators of the effects of I Move were found.

**Conclusions:**

The results suggest that web-based computer tailored physical activity interventions might best include elements based on both self-determination theory/motivational interviewing and traditional health behavioral theories. To be more precise, it is arguable that the focus of the theoretical foundations, used in new web-based PA interventions should depend on the intended program outcome. In order to draw firm conclusions, however, more research on the effects of self-determination theory and motivational interviewing in web-based physical activity promotion is needed.

**Trial registration:**

Dutch Trial Register NTR4129

## Background

Regular physical activity (PA) is highly beneficial for health, and decreases the risk of many adverse health conditions such as coronary heart disease, type 2 diabetes and breast and colon cancer [1]. Unfortunately, large parts of the world populations are insufficiently physically active [2] thus making the promotion of PA a public health priority [3, 4]. Previous research has shown that intensive and repeated counseling by health care professionals is effective in getting individuals to increase their PA level [5–7]. However, face-to-face PA counseling interventions are often too expensive to be implemented on a population level [8, 9].

Web-based computer tailored PA interventions might be a plausible alternative to individual PA counseling [10]. These interventions provide individualized feedback that matches personal characteristics and needs [10–12]. Delivery via the internet makes it possible to reach large numbers of people for relatively low costs [10, 11, 13, 14]. Recent meta-analyses show that web-based computer tailored PA interventions are capable of producing modest but significant short-term increases in PA [15, 16]. However, several studies show that these short-term effects tend to diminish or extinguish as follow-up up time increases [15, 16].

To date, web-based computer tailored PA interventions have typically been based on traditional health behavior theories such as social cognitive theory (SCT), self-regulation theory (SRT), the trans-theoretical model (TTM) and the theory of planned behavior (TPB) [12, 15, 17]. Interventions of this type, hereafter referred to as ‘traditional interventions’, make use of theoretical constructs such as stages of change, modeling, attitude and self-efficacy [15]. Recent research on determinants of sustained PA behavior, however, shows another theoretical construct to be essential: autonomous motivation [18–21]. Substantial evidence suggests that having higher autonomous motivation makes an individual more likely to persist with a PA routine [22]. Although the concept of autonomous motivation does not feature explicitly in SCT, SRT, TTM or TPB, it is central to self-determination theory (SDT) and motivational interviewing (MI) [23, 24]. Applying the principles of SDT and MI in web-based computer-tailored PA interventions could be a promising improvement for these interventions, and could possibly be more effective in promoting sustained PA behavior than traditional web-based computer tailored PA interventions [25].

In order to gain more insight into the effectiveness and feasibility of applying SDT and MI in web-based PA promotion, we systematically developed I Move, a web-based computer tailored PA intervention, grounded in SDT and MI [23, 24, 26]. I Move was found to be effective in significantly increasing PA behavior in the short term (3–6 months after baseline), compared to a no-intervention control condition (Friederichs S, Oenema A, Bolman C, Lechner L: Effects of motivational interviewing and self-determination theory in a web-based computer tailored physical activity intervention: a randomized controlled trial, submitted). The short-term effects of I Move on PA behavior were comparable to the short-term effects of Active Plus (a traditional web-based computer tailored PA intervention). Autonomous motivation, however, may be especially important for sustained changes in PA behavior. Differences in effects on PA between I Move and Active Plus could therefore become more pronounced in the long-term. In the current paper, we aim to assess how the 6-month effects of I Move on PA behavior evolve over time (until 12 months from baseline), compared to Active Plus, and compared to a no-intervention control group. First, however, we provide additional background on SDT and MI.

### Self-determination theory & motivational interviewing

SDT is a comprehensive theory of behavioral motivation [24, 27, 28], which has proven to be particularly useful in the context of PA research, both for accounting for patterns of PA behavior and for informing the development of interventions for promoting PA [22, 29]. Central to this theory is the difference between autonomous and controlled motivation. Both autonomous and controlled motivation influence behavior, but they each lead to a different outcome, with autonomous motivation leading to greater commitment and long-standing maintenance of behavior [24, 27, 28, 30]. SDT posits that individuals are more likely to exhibit autonomous motivation when three basic psychological needs are supported: autonomy (i.e. the need to feel that one can choose one’s behaviors), competence (the need to feel competent and confident) and relatedness (the need to feel connected to and understood by others) [24, 27, 28]. Motivational interviewing (MI) is defined as “a collaborative conversation style for strengthening a person’s own motivation and commitment to change” [23]. Several researchers have argued that the specific client-centered communication skills used in motivational interviewing (MI) can be used to support client’s basic psychological needs [29, 31–33].

In recent years, numerous PA counseling interventions that combine the theoretical framework of SDT with the practical strategies from MI have been developed and evaluated in randomized controlled trials [19, 34–43]. In general, these interventions are effective in promoting a sustained increase in PA. As discussed above, however, face-to-face PA counseling interventions are often too expensive to be implemented on a large scale [8, 9]. Web-based computer tailored PA interventions grounded in SDT and using the communication style and principles from MI, may be promising for promoting sustained PA behavior on the population level at relatively low costs [25]. To our knowledge, however, no studies have yet evaluated the long term effects of SDT and MI in web-based PA promotion.

### Research objectives

Our main objective in the current study was to evaluate the long-term effectiveness (12 months from baseline) of I Move (a web-based computer tailored PA intervention aimed at adults, based on SDT and MI). To this end, we compared I Move to a traditional web-based computer tailored PA intervention (Active Plus), and to a no-intervention control group. It was hypothesized that at 12 months from baseline, the effects of I Move would be better retained compared to the effects of Active Plus.

Moderator analyses can identify the participant subgroups in which an intervention exerts the greatest effects, enabling a more in-depth view on impact of the intervention. Such results can potentially yield recommendations for future intervention adaptation and development [44]. Previous studies on tailored PA interventions have shown that participant characteristics (such as age and gender and the intention to be sufficiently physically active) can moderate the effects of tailored PA interventions [45–52]. The short-term effects of I Move on PA at 6 months were moderated by gender, age and relational status; the effects of I Move were more pronounced in male participants, participants younger than 47, and participants who were single. Therefore, as a secondary objective the present study aimed to identify participant characteristics that moderate the long term effects of I Move and Active Plus.

## Method

For the purpose of this study, we conducted a randomized controlled trial (RCT), which was registered with the Dutch Trial Register (NTR4129).

### Participants and procedure

Individuals between 18 and 70 years old, who did not have a condition that seriously impedes their ability to be physically active, who did not take part in one of the pilot studies [53, 54], and who were less physically active than 5 days per week for 60 min per day were considered for participation [26]. A power calculation (ES = .25; power = .80), showed that data from 600 participants would be needed for this study. Based on an expected dropout of 40-70 % [55, 56], we needed to enroll 2,000 respondents in the study.

**Fig. 1:**
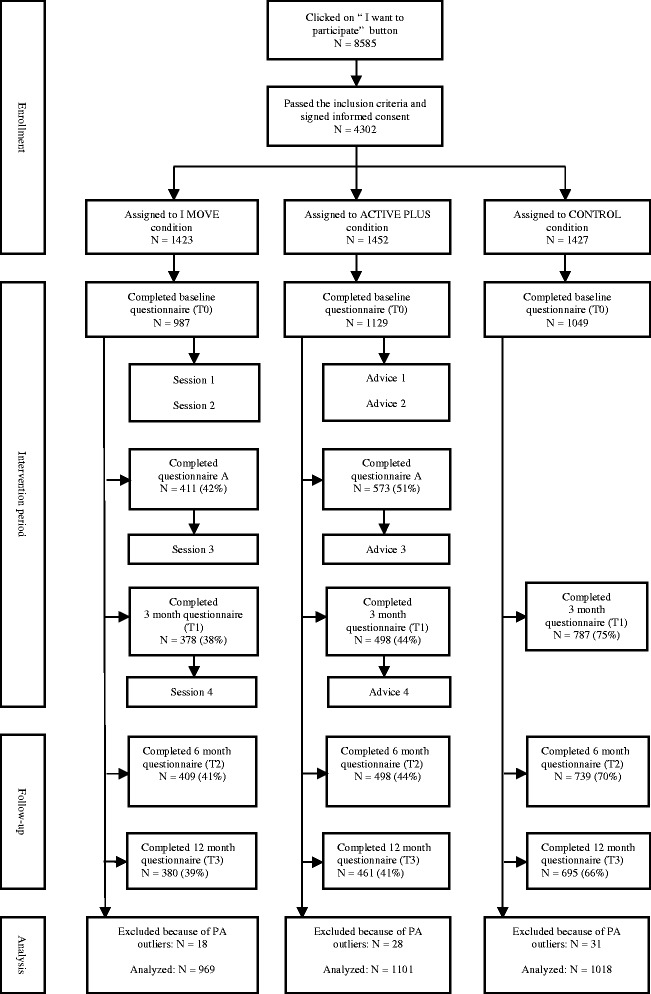
Flow of participants

The study procedure and flow of participants is shown in Fig. 1. From September to December 2013, participants were recruited by means of advertisements (containing a link to the study website) in national newspapers, social media, and via an online panel. At the study website, individuals were given the option to participate in the study by clicking on the “*I want to participate*” button. After passing through the inclusion questions and giving informed consent, participants were randomized into one of the three research conditions (1:1:1) by means of a digital randomizer built into the website, and were asked to complete the baseline questionnaire. The three research conditions in this study are: 1) the I Move condition; participants in this condition received I Move, a web-based computer tailored PA intervention based on MI and SDT [26], 2) the Active Plus condition; participants in this condition received Active Plus, a traditional web-based computer tailored PA intervention, based on traditional health behavioral theories such as TPB, SCT, SRT and TTM [57, 58] and 3) the control condition, a waiting list control group.

Measurements were taken at baseline, and at 3, 6 and 12 months from baseline. All measurements were taken by web-based questionnaires via the study website. Participants from the two intervention conditions were also asked to complete a questionnaire at 6 weeks from baseline. This questionnaire contained questions that are used to tailor the intervention content, as well as questions on process evaluation. In order to decrease attrition, 10 prizes of €50 were raffled off among those participants who completed each questionnaire [59]. Among those participants who completed all questionnaires, two tablet computers were raffled off.

### Ethics, consent and permissions

The Medical Ethics Committee of Atrium-Orbis-Zuyd approved the RCT. Before being able to participate in the study, all potential participants were asked to give informed consent.

### Interventions

I Move is a web-based computer tailored PA intervention, aimed at increasing and maintaining PA among adults [26]. The intervention is based on the theoretical insights of SDT and the practical applications of MI. Since developing interventions in a systematically planned way increases the likelihood of effectiveness [60], we used the Intervention Mapping protocol [61] to develop I Move.

I Move entails four automated text-based sessions. During these sessions, participants answer several questions (either by typing in their answer or choosing their answer from a list). In between those questions they receive tailored feedback text messages and tailored follow-up questions. As such, a motivational dialogue is simulated between the intervention and the participant. Alongside the text-based sections, at regular times during the intervention, participants are offered the option of watching short videos (starring a program host, a PA-expert and four allegedly former intervention participants). Session 1 starts with an introduction, and after that several topics such as the participant’s current PA, how important he/she thinks it is to increase his/her PA level, and how confident he/she is with regards to becoming more physically active are discussed. Participants can choose to make an action plan to become more active. Three weeks and six weeks later, participants receive an email invitation for session 2 and 3, respectively. Session 2 and 3 address in more detail the topics of perceived importance of and perceived confidence in becoming more physically active. Participants are given the opportunity to evaluate and adjust their plans, and to formulate coping plans. During session 3, participants receive feedback on their current PA level as compared to their PA level at baseline. Three months after session 1, participants receive an email invitation for session 4. In this session, participants are given the option to choose which parts of the session they want to go through (ipsative feedback on PA, long-term motivation and confidence). The day after having completed an intervention session, participants receive a PDF file by email, containing a summary of the session content. For more detailed information on the content and basis of I Move, please see the separate design paper [26].

Active Plus is a systematically developed, theory- and evidence-based web-based computer tailored PA intervention [57, 58]. This intervention is predominantly founded on traditional health behavior change theories such as TPB, SCT, SRT and TTM. The original Active Plus intervention encompasses three rounds of tailored advice [57, 58]. In order to make Active Plus optimally comparable to I Move (which contains four intervention moments), a fourth tailored advice was added to Active Plus. Furthermore, since Active Plus was originally designed for individuals over 50 years of age, we adapted the content slightly in order to make it appropriate for the general adult population.

**Fig. 2:**
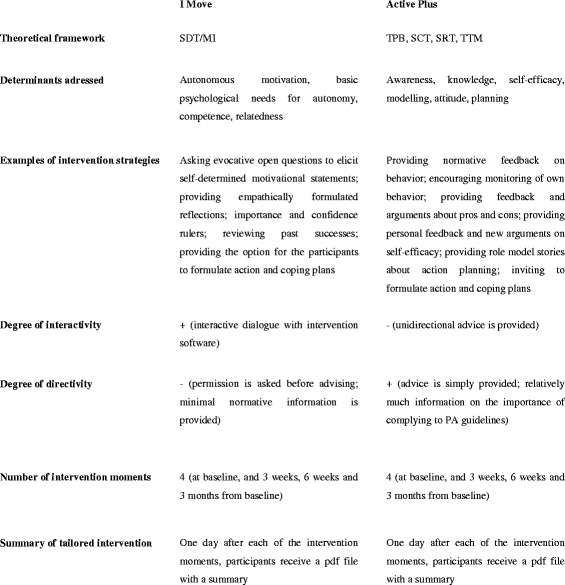
General similarities and differences between both interventions

An important difference between I Move and Active Plus concerns the degree of interactivity. In Active Plus, participants are first asked to complete a questionnaire on all the relevant psychological constructs, and then they the complete advice. I Move is designed to simulate a conversation with the participant by means of an interactive question-feedback approach. Hence, the participant is asked to actively participate in the intervention by responding to the questions, and reading the feedback messages. In addition, several other differences between the interventions exist, many of which are related to the fact that I Move provides more autonomy support. For example, before being provided with information on PA, participants in I Move are first asked whether they would like to read some more about the beneficial effects of PA, while in Active Plus no permission is asked, and information is simply provided. In Fig. 2, the most important similarities and differences between the interventions are summarized.

### Questionnaire

#### Demographics

At baseline, age, gender, weight and height (combined to BMI), relational status and highest completed educational level were assessed. Educational level was categorized into high (higher vocational school or university level) and low to medium (elementary education, medium general secondary education, preparatory vocational school, lower vocational school, higher general secondary education, preparatory academic education, and medium vocational school), according to the Dutch educational system.

#### PA intention

As intention to become sufficiently active can be an important predictor of dropout in web-based PA interventions [56], this was measured at baseline. Intention to become sufficiently active was measured with 3 items (e.g. ‘Are you planning to be sufficiently physically active?’ Definitely not (1) – Yes, definitely (10)). Participants were informed that sufficient physical activity is at least 30 min of moderate-intensity PA at least 5 days a week.

#### PA level

PA behavior was measured at baseline, 3, 6 and 12 months using the validated self-administered Dutch Short Questionnaire to Assess Health Enhancing Physical Activity (SQUASH) [62]. All questions in the SQUASH were asked for a typical week in the past month. Although objective observation might be more accurate, self-report is often the most feasible method when assessing PA in large-scale studies because of the convenience and low costs [63]. Compared to self-report, measuring PA levels with accelerometers is rather intrusive, which does not fit well with the accessible nature of a web-based intervention.

Total weekly minutes of moderate to vigorous PA (MVPA) was calculated by multiplying the frequency (how many days per week), and duration (how many hours and minutes per day) of leisure and transport walking, leisure and transport cycling, sports, gardening, household chores and odd jobs performed with moderate or vigorous intensity. The relative validity (*r*_spearman_ = 0.45; 95 % CI = 0.17–0.66) and reproducibility (*r*_spearman_ = 0.58; 95 % CI = 0.36–0.74) of the SQUASH are reasonable for the general adult population [65].

Total weekly days with more than 30 min PA were measured by a single item: ‘How many days per week are you, in total, moderately physically active by undertaking, for example, brisk walking, cycling, chores, gardening, sports, or other physical activities for at least 30 min?’ Prior research provided support for the validity and reliability of single-item self-reports of PA [64, 65] and several studies found the single item PA measure to be among the most accurate PA questionnaires, when compared to accelerometer output [66, 67].

### Statistical analysis

All analyses were conducted using SPSS version 22. As instructed by the SQUASH manual, participants who reported over 6,720 min of PA per week were excluded from all analyses because being physically active more than 16 h per day for 7 days per week was assumed to be impossible [62].

Pearson’s correlation was calculated between the baseline values of weekly minutes of MVPA and weekly days with more than 30 min PA. Chi-square tests and one-way analyses of variance (ANOVAs) were conducted to assess potential baseline differences among the research conditions. Logistic regression analyses were performed to identify predictors of dropout at the 12-month questionnaire.

#### Main outcome analyses

First, actual PA values (mean and SD) were calculated for each research condition at each timepoint. To evaluate the effects of both interventions on PA, multilevel linear regression analyses with two levels: *time* was nested within *participant*. Research condition was coded into two dummy variables, dummyIMOVE and dummyACTIVEPLUS, using the control condition as reference. Separate analyses were conducted for both PA outcome measures (total weekly minutes of MVPA, and weekly days with ≥ 30 min PA). In both analyses, time, dummyIMOVE, dummyACTIVEPLUS, dummyIMOVE*time and dummyACTIVEPLUS*time were entered as independent variables. Variables included as covariates were: baseline PA value (minutes and days), gender, age, educational level, relational status, BMI and baseline intention to be sufficiently physically active. To correct for bias because of selective drop-out, analyses were conducted using the whole dataset, including missing data (intention-to-treat). Conducting multilevel analyses on an incomplete dataset including missing data has been shown to give better estimations than using multiple imputation [68]. Due to the flexible treatment of the time predictor, multilevel analyses can make use of all available data in the estimation of model parameters. As such, research participants with only baseline data can be included in an analysis and contribute to the estimation of model parameters [68]. Aside from the intention-to-treat analyses, completers-only analyses were conducted (including only those participants who provided 12 month PA data).

Cohen’s *d* effect sizes (ESs) were computed for both measurement moments (6 months from baseline and 12 months from baseline) based on the unstandardized regression coefficients and the pooled standard deviation of the outcome measure (at 6 months from baseline and 12 months from baseline, respectively) [69]. Effect sizes of 0.15, 0.20 and 0.25, respectively, were considered small, medium and large [70].

#### Moderator analyses

In order to assess whether the long-term effects of both interventions on PA were more pronounced in specific subgroups of participants, additional moderator analyses were conducted. In these moderator analyses, only the 12-month follow-up measurements were included. Interaction terms between the intervention dummies and gender, age, educational level, relational status, BMI and intention to be sufficiently physically active were assessed. Separate moderation analyses were performed for each of the user characteristics. If an interaction term had a *p*-value < .1 this interaction was decomposed (using a median split in the case of a continuous variable) and subgroup analyses were performed [71–75].

## Results

In Fig. 1, an overview of the flow of participants in the study is presented. A significant correlation was found between baseline values of weekly minutes of MVPA and weekly days with more than 30 min PA (r .214; *p* < .001). As shown in Table 1, no baseline differences on key characteristics were found between the three study conditions. Dropout analysis showed that the lower participants’ age (B = 0.018 ± 0.003; *p* < .001) and the lower their intention to be sufficiently physically active (B = 0.090 ± 0.022; *p* < .001), the less likely they were to complete the 12 month PA questionnaire. In addition, the more weekly minutes of MVPA reported at baseline (B < 0.001 ± 0.000; *p* < .001) and the fewer weekly days with ≥ 30 min PA reported at baseline (B = 0.080 ± 0.024; *p* = .001), the less likely participants were to complete the 12 month PA questionnaire. Finally, participants were less likely to complete the 12 month PA questionnaire if they were married or living together (OR = 1.32; 95 % CI = 1.11-1.57; *p* = .002), if they had a lower education level (OR = 1.22; 95 % CI = 1.05-1.43; *p* = .011) and if they were randomized in one of the intervention conditions (OR_IMove_ = 3.28; 95 % CI = 2.72-3.95; *p* < .001; OR_ActivePlus_ = 3.00; 95 % CI = 2.51-3.60; *p* < .001). These predictors of dropout were included in the effect analyses as covariates.

**Table 1:** Baseline characteristics

	I Move	Active Plus	No intervention	*F/χ* ^*2*^ (df = 2)
	n = 969	n = 1101	n = 1018	
Gender: % male	30.2 %	31.4 %	30.8 %	0.34
Age	44.99 ± 13.12	45.25 ± 12.67	44.48 ± 12.69	0.97
Relational status: % married/living together	74.4 %	74.3 %	73.9 %	0.09
Education: % high education	63.2 %	61.4 %	61.0 %	1.11
BMI	26.16 ± 4.78	26.06 ± 5.22	25.81 ± 4.51	1.33
Physical activity, weekly minutes	510 ± 573	479 ± 541	501 ± 554	0.89
Physical activity, weekly days	3.09 ± 1.70	3.09 ± 1.65	3.14 ± 1.70	0.16
Intention to be sufficiently physically active	7.07 ± 1.89	7.16 ± 1.81	7.16 ± 1.88	0.71

### Intervention effects

Table 2 shows the actual PA values for the three research conditions at all time points. In Table 3, the results of the intention-to-treat and completers-only analyses are shown. As can be seen in this table, the results from intention-to-treat and the completers-only analyses are highly similar. The results will be further discussed below.

**Table 2:** Absolute PA levels at baseline, 6 months and 12 months

Weekly minutes of MVPA			
	Baseline	6 months	12 months
I Move	510 ± 573	640 ± 647	618 ± 625
Active Plus	479 ± 541	637 ± 665	540 ± 501
Control	501 ± 554	558 ± 581	536 ± 512
Weekly days with ≥ 30 min PA			
	Baseline	6 months	12 months
I Move	3.09 ± 1.70	4.06 ± 1.88	4.02 ± 1.86
Active Plus	3.09 ± 1.65	4.35 ± 1.81	4.04 ± 2.02
Control	3.13 ± 1.70	3.73 ± 1.89	3.84 ± 1.88

**Table 3:** Intervention effects on weekly minutes of MVPA and weekly days with ≥ 30 minutes PA

	Intention-To-Treat						Completers-Only					
	Weekly minutes of MVPA						Weekly minutes of MVPA					
	B	*p*	ES	B	*p*	ES	B	*p*	ES	B	*p*	ES
*Differences in change*	*6 months after baseline*			*12 months after baseline*			*6 months after baseline*			*12 months after baseline*		
IMOVE vs CONTROL	76.22	.015	.13	70.65	.030	.13	95.92	.004	.17	78.16	.015	.13
ACTIVEPLUS vs CONTROL	87.15	.003	.14	17.54	.567	.03	90.14	.005	.18	18.92	.531	.03
IMOVE vs ACTIVE PLUS	−10.94	.745	−.02	53.11	.132	.09	5.77	.875	.01	59.24	.089	.09
	Weekly days with ≥ 30 min PA						Weekly days with ≥ 30 min PA					
	B	*p*	ES	B	*p*	ES	B	*p*	ES	B	*p*	ES
*Differences in change*	*6 months after baseline*			*12 months after baseline*			*6 months after baseline*			*12 months after baseline*		
IMOVE vs CONTROL	0.32	.001	.17	0.16	.110	.09	0.32	.002	.17	0.16	.112	.09
ACTIVEPLUS vs CONTROL	0.60	< .001	.32	0.22	.023	.11	0.62	< .001	.32	0.22	.020	.12
IMOVE vs ACTIVE PLUS	−0.28	.006	−.15	−0.05	.615	−.03	−0.30	.009	−.15	−0.06	.577	−.03

### Intervention effects on weekly minutes of MVPA: intention-to-treat

In these analyses, I Move had a significant effect on weekly minutes of MVPA at 6 months from baseline; participants from the I Move condition increased their weekly minutes of MVPA by 76 min, compared to the control condition (B = 76.22; *p* = .015; ES = .13). Active Plus also achieved a significant effect on this outcome at 6 months; participants from the Active Plus condition increased their weekly minutes of MVPA by 87 min, compared to the control condition (B = 87.15; *p* = .003; ES = .14). The effects of both interventions on weekly minutes of MVPA at 6 months did not differ significantly from each other (B = −10.94; *p* = .745; ES = −.02). At 12 months from baseline, I Move had a significant effect on weekly minutes of MVPA; participants from the I Move condition increased their weekly minutes of MVPA by 71 min, compared to the control condition (B = 70.65; *p* = .030; ES = .13). The Active Plus condition did not have a significant effect on this outcome at 12 months (B = 17.54; *p* = .576; ES = .03). However, I Move was not significantly more effective in increasing weekly minutes of MVPA at 12 months, compared to Active Plus (B = 53.11; *p* = .132; ES = .09).

### Intervention effects on weekly minutes of MVPA: completers-only

In these analyses, I Move had a significant effect on weekly minutes of MVPA at 6 months from baseline; participants from the I Move condition increased their weekly minutes of MVPA by 96 min, compared to the control condition (B = 95.92; *p* = .004; ES = .17). Active Plus also achieved a significant effect on this outcome at 6 months; participants from the Active Plus condition increased their weekly minutes of MVPA by 90 min, compared to the control condition (B = 90.14; *p* = .005; ES = .18). The effects of both interventions on weekly minutes of MVPA at 6 months did not differ significantly from each other (B = 5.77; *p* = .875; ES = .01). At 12 months from baseline, I Move had a significant effect on weekly minutes of MVPA; participants from the I Move condition increased their weekly minutes of MVPA by 78 min, compared to the control condition (B = 78.16; *p* = .015; ES = .13). The Active Plus condition did not have a significant effect on this outcome at 12 months (B = 18.92; *p* = .531; ES = .03). However, I Move was not significantly more effective in increasing weekly minutes of MVPA at 12 months, compared to Active Plus (B = 59.24; *p* = .089; ES = .09).

### Intervention effects on weekly days with ≥ 30 min PA: intention-to-treat

In these analyses, I Move had a significant effect on weekly days with ≥ 30 min PA at 6 months from baseline; participants from the I Move condition increased their weekly days with ≥ 30 min PA by 0.3 days, compared to the control group (B = 0.32; *p* = .001; ES = .17). The Active Plus condition also achieved a significant effect on this outcome at 6 months; participants from the Active Plus condition increased their weekly days with ≥ 30 min PA by 0.6 days, compared to the control group (B = 0.60; *p* < .001; ES = .32). At 6 months, the effect of Active Plus on weekly days with ≥ 30 min PA was significantly higher than the effect of I Move; participants from the Active Plus condition increased their weekly days with ≥ 30 min PA by 0.3 days, compared to participants from the I Move condition (B = 0.28; *p* = .006; ES = .15). At 12 months from baseline, I Move did not have a significant effect on weekly days with ≥ 30 min PA (B = 0.16; *p* = .110; ES = .09). The Active Plus condition had a significant effect on this outcome at 12 months; participants from the Active Plus condition increased their weekly days with ≥ 30 min PA by 0.2 days, compared to the control condition (B = 0.22; *p* = .023; ES = .11). However, Active Plus was not significantly more effective in increasing weekly days with ≥ 30 min PA at 12 months, compared to I Move (B = 0.05; *p* = .615; ES = .03).

### Intervention effects on weekly days with ≥ 30 min PA: completers-only

In these analyses, I Move had a significant effect on weekly days with ≥ 30 min PA at 6 months from baseline; participants from the I Move condition increased their weekly days with ≥ 30 min PA by 0.3 days, compared to the control group (B = 0.32; *p* = .001; ES = .17). The Active Plus condition also achieved a significant effect on this outcome at 6 months; participants from the Active Plus condition increased their weekly days with ≥ 30 min PA by 0.6 days, compared to the control group (B = 0.62; *p* < .001; ES = .32). At 6 months, the effect of Active Plus on weekly days with ≥ 30 min PA was significantly higher than the effect of I Move; participants from the Active Plus condition increased their weekly days with ≥ 30 min PA by 0.3 days, compared to participants from the I Move condition (B = 0.30; *p* = .009; ES = .15). At 12 months from baseline, I Move did not have a significant effect on weekly days with ≥ 30 min PA (B = 0.16; *p* = .112; ES = .09). The Active Plus condition had a significant effect on this outcome at 12 months; participants from the Active Plus condition increased their weekly days with ≥ 30 min PA by 0.2 days, compared to the control condition (B = 0.22; *p* = .020; ES = .12). However, Active Plus was not significantly more effective in increasing weekly days with ≥ 30 min PA at 12 months, compared to I Move (B = 0.06; *p* = .577; ES = .03).

### Moderators

Age moderated the 12 month effect of Active Plus on weekly days with ≥ 30 min PA (*p* = .040). Active Plus was more effective in increasing days with ≥ 30 min PA in participants aged 47–70 (B = 0.368; *p* = .008; ES = .19) than in participants aged 18–46 (B = 0.077; *p* = .583; ES = .04). No additional significant moderators of the intervention effects were found.

## Discussion

At 12 months from baseline, I Move was found to be effective in increasing weekly minutes of MVPA (ES = .13; *p* = .030), while Active Plus was not (ES = .03; *p* = .567). In contrast, Active Plus was found to be effective in increasing weekly days with ≥ 30 min PA at 12 months (ES = .11; *p* = .023), while I Move was not (ES = .09; *p* = .110).

The difference between the 12-month effects of the interventions on weekly minutes of MVPA was not statistically significant (ES = .09; *p* = .132). However, this difference (about one hour of weekly MVPA) might still be of practical importance [56]. At 6 months from baseline, both interventions yielded a comparable effect on weekly minutes of MVPA per week, while at 12 months from baseline the effect of Active Plus had mostly disappeared (ES = .03; *p* = .567), and the positive effect of I Move was sustained (ES = .13; *p* = .030). Although more evidence is needed in order to draw firm conclusions, this trend might be supportive for the use of SDT and MI in web-based CT in order to achieve maintained PA increase. The client-centered approach, as operated in I Move, may have prompted participants to adopt PA behavior for self-determined reasons. As discussed earlier, autonomous motivation facilitates long-standing maintenance of behavior which may partly explain the long-term effectiveness of I Move on this outcome [24, 27, 28, 30]. Then again, the 12 month effect of I Move on weekly minutes of MVPA is not overwhelming. The long-term effect size of the I Move intervention on this outcome was .13 (9 months after the end of the intervention), while in a meta-review on web-based PA interventions, the overall mean effect was .11. (6 months after the end of the intervention) [15]. Therefore, I Move may need to be further improved in order to optimize its long-term intervention effect.

No statistically significant differences were found for the interventions with regard to weekly days with ≥ 30 min outcome at 12 months (ES = .03; *p* = .615). Even so, Active Plus was effective in increasing weekly days with ≥ 30 min PA (ES = .11; *p* = .023), while I Move was not (ES = .091; *p* = .110). This may be related to the fact that I Move does not provide much information on PA guidelines. According to the principles of SDT and MI, participants in I Move were encouraged to set their *own* PA norms/goals (which are not necessarily in line with the PA guidelines).

Additional moderator analyses showed that the long-term effects of I Move were not moderated by any participant characteristics; this is supportive of the degree and quality of tailoring in this intervention. The absence of any effect moderators indicates that I Move probably can be effective in increasing weekly minutes of MVPA in different subgroups; for instance in participants with different levels of education, and in different age groups. Active Plus was found to be more effective in increasing days with ≥ 30 min PA in participants aged 47–70 than in participants aged 18–46. This finding might be explained by the fact that Active Plus was originally designed for individuals over 50 years. The intervention content was adapted in order to make it suitable for the general adult population [57, 58]. However it is possible that the intervention was still more appealing and motivating for older participants.

### Strengths and limitations

The study reported in this paper is the first to examine the long-term effects of a web-based PA intervention based on SDT and MI. Strengths of this study include the strong randomized controlled design with a large sample size, and no differences between the study conditions at baseline. In addition, self-reported physical activity was measured using a validated measurement instrument [62].

Despite these strengths, the study has some limitations that should be acknowledged. First, although we did our best to develop I Move as congruent as possible to MI and SDT, important differences exist between I Move and MI or SDT-based counseling in a face-to-face context. For instance, compared to I Move, a real human counselor is better able to convey empathy and to react to very subtle expressions of motivation. This leaves room for improvement and further development. In the future, more sophisticated techniques may be able to improve the ability of web-based PA interventions to respond empathically. Second, the fact that the interventions in our studies differ from each other with regard to both theoretical basis and degree of interactivity represents a potential methodological weakness. One could argue, however, that using an interactive approach, as was done for the I Move intervention, is inherent to developing an intervention based on MI and SDT, while this is less obvious when working with traditional health behavioral theories. Still, it would be relevant for future studies on tailored interventions to assess the influence of theoretical framework and degree of interactivity separately. Third, the attrition was considerable. Unfortunately, attrition is very common in studies on eHealth and web-based interventions [76]. Attrition was higher in the intervention conditions compared to the control condition. This is in line with the findings from a recent review on differential attrition in health behavior change interventions [77]. In this study, we aimed to handle the missing data in the most accurate way possible by conducting the main analyses using multilevel linear regression [68]. Indeed, applying multilevel analyses to an incomplete dataset has been shown to give better estimations than using multiple imputation [68]. Fourth, the results of this study may be influenced by selective drop-out (e.g., younger participants dropped out more frequently than did older participants). By including all drop-out predictors in the regression analyses as covariates, and by analyzing the total dataset including missing values, we aimed to account for selective drop-out as much as possible [68]. Fifth, it should be noted that female and highly educated participants were overrepresented in the research population. By conducting moderator analyses, however, we aimed to evaluate whether gender, educational level, and other individual participant characteristics influenced intervention outcomes; we found this not to be the case for I Move. Finally, PA behavior was assessed using self-report questionnaires and although the SQUASH has reasonable reproducibility and relatively good validity [62] a risk remains that the data were subject to biases, for example over-estimation of PA or socially desirable responding [78, 79]. In our study, there clearly was over-reporting of PA behavior (at baseline, participants reported to be physically active for ± 500 min per week). However, since over-reporting was present in all three research condition (including the control condition), it was possible to assess changes in PA behavior over time in a reliable way. Still, future studies on web-based PA interventions should include a more objective measure of PA behavior [80, 81]. Using a more elaborate PA measure would allow to better distinguish between PA at weekdays and PA at weekend days, which could provide useful information on PA patterns throughout the week. However, assessing PA with even more details and additional questions could also result in even more over reporting of PA.

## Conclusions

The present study is the first to evaluate SDT and MI in a web-based computer tailored PA intervention. In this study, I Move was effective in increasing weekly minutes of MVPA at 12 months from baseline, while the effect of Active Plus on this outcome disappeared. This finding provides support for the use of SDT and MI in web-based computer tailored PA interventions. However, Active Plus was found to be effective in increasing weekly days with ≥ 30 min PA at 12 months, while I Move was not. Together these results suggest that web-based computer tailored PA interventions might best include elements based on both SDT/MI and traditional health behavioral theories. To be more precise, it is arguable that the focus of the theoretical foundations, used in new web-based PA interventions should depend on the intended program outcome. If the intended program outcome is to get individuals to comply with PA guidelines, an emphasis on traditional health behavioral theories might be most suitable. If the intended program outcome is to increase overall PA behavior (without taking into account PA guidelines), making strong use of MI and SDT might be more appropriate. However, in order to draw firm conclusions, more research should be done on the effects of SDT and MI in web-based PA promotion. Future research should also assess the working mechanism underpinning the long-term effects of this type of intervention, and whether or not these effects are mediated by an increase in autonomous motivation.

## Abbreviations

ANOVA: Analysis of variance
ES: Effect size
MI: Motivational interviewing
MVPA: Moderate to vigorous physical activity
PA: Physical activity
RCT: Randomized controlled trial
SCT: Social cognitive theory
SDT: Self-determination theory
SRT: Self-regulation theory
TPB: Theory of planned behavior
TTM: Trans-theoretical model
